# Chlorophyll *b*—An Essence of Plant Photosynthesis

**DOI:** 10.3390/plants15131969

**Published:** 2026-06-26

**Authors:** John Kenneth Hoober, Laura L. Eggink, Daniel-Paul Bednarik, Steffen Reinbothe

**Affiliations:** 1 Wild Boar Biosciences, LLC, 1615 W. University Drive, Suite 132, Tempe, AZ 85281, USA; leggink@wildboarbio.com (L.L.E.); dbednarik@gmail.com (D.-P.B.); 2 Laboratoire de Génétique Moléculaire des Plantes, PAVAL, Université Grenoble Alpes, 38400 Grenoble, France

**Keywords:** Chlorophyllide (Chlide) *b*, Chlide *a* oxygenase, phenanthroline, redox potential, tyrosyl radical, chloroplast development, Chlamydomonas, Arabidopsis

## Abstract

Chlorophyll (Chl) *b* is crucial for assembly of the light-harvesting antennae that are required for optimal photosynthetic activity in plants and green algae. Synthesis of its precursor, chlorophyllide (Chlide) *b*, is catalyzed by Chlide *a* oxygenase (CAO), which contains a stable tyrosyl radical. Studies with the model organism *Chlamydomonas reinhardtii y-1* suggested that protochlorophyllide (Pchlide) *a* is a substrate for the enzyme in the dark when a ‘cofactor’ is present to form a heterodimer, which apparently decreases the redox potential of Pchlide *a*. Data described in the literature are consistent with reduction in the redox potential of Chlide *a* by dimerization, which produces a substrate that allows rapid synthesis and accumulation of Chl *b* during chloroplast development in oxygenic photosynthetic organisms. In this article, we provide an emerging perspective on CAO’s structure, its assumed radical-mediated catalytic mechanism, and its role *in planta*.

## 1. Introduction

### 1.1. Primary Function of Chl b

Life on this planet is supported by the conversion of light energy from the sun into chemical energy during photosynthesis. The primary step in the conversion of energy is light-induced charge separation that is catalyzed by two distinct pigment-protein complexes designated photosystem (PS) I and PS II in thylakoid membranes. Both photosystems contain in their reaction centers a special pair of chlorophyll (Chl) *a*-type molecules, named P700 and P680, respectively, as well as associated Chl *a*-binding proteins. Surrounding the photosystems are Chl *a* and Chl *b* molecules that are bound to light-harvesting complex apoproteins (LHCPs) and provide the necessary function as antennae for light capture [1]. Prior to their esterification with geranylgeranyl pyrophosphate, chlorophyllide (Chlide) *a* is converted to Chlide *b.* A consequence of this structural modification—formation of a 7-formyl group from the 7-methyl group—is a shift in the absorbance spectrum that broadens the ability of plants to harvest light. In autotrophic plant cells, Chl *b* is essential for the assembly of stable, functional light-harvesting complexes (LHCs). Plants that lack Chl *b* because of loss-of-function mutations are markedly deficient in LHCs in thylakoid membranes, have pale green or yellow–green leaves, and are designated *chlorina* mutants.

**Figure 1:**
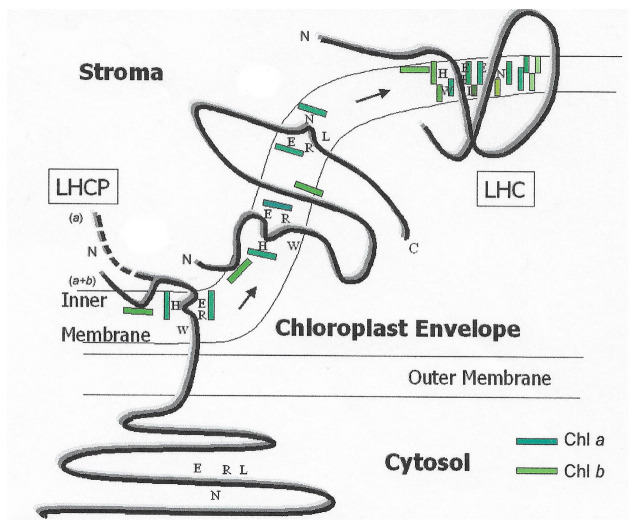
Assembly of the light-harvesting complex (LHC) in the inner membrane of the chloroplast envelope. The initial stage involves sufficient import of the NH_2_-terminal domain of the precursors of the LHCPs for the transit sequence to be removed by stromal proteases. One Chl *b* and two Chl *a* molecules bind to the first membrane-spanning segment to anchor the proteins, while the remainder is imported into the membrane. A motif in the first membrane-spanning helix, which is repeated in the third membrane-spanning segment, with asparagine (N) replacing histidine (H), binds two molecules of Chl *a*. Finally, the proteins fold with the full complement of Chl and carotenoids.

LHCPs are synthesized in the cytosol of plant cells as precursors that contain an NH_2_-terminal extension designated the ‘transit sequence.’ After synthesis, the transit sequence guides the proteins through translocon complexes into the plastid envelope inner membrane, where the final steps in Chl synthesis occur. The transit sequence is then removed by proteases in the plastid stroma. The EVIHxRW motif within the first membrane-spanning helix of the major LHCPs binds two molecules of Chl *a*, one to the imidazole group of histidine (H) and the second to the glutamate (E)-arginine (R) ion pair [2]. Chl *b* does not bind to this motif, but the 7-formyl group strengthens the Lewis acid character of the central Mg^2+^ and allows Chl *b* to bind to strong Lewis bases such as the peptide bond carbonyl oxygen. A peptide-bond oxygen is contributed by a tyrosine residue near the NH_2_-terminus and is not H-bonded because of a proline residue one helical turn upstream [3]. The binding of Chl molecules to these three sites allows the remainder of the proteins to enter the envelope inner membrane and assemble the light-harvesting complexes (LHCs) (Figure 1).

In our studies of LHC assembly in the model alga *Chlamydomonas reinhardtii y-1*, the genes encoding the LHCPs were transcribed at 38 °C, but the proteins were not synthesized at a significant rate in the dark [4]. Interestingly, the LHCPs were synthesized in the dark as much as in the light in the Chl *b*-less, *cbn1-113 arg2 mt*^+^ mutant of the alga, but the proteins were detected by immunoelectron microscopy in the cytosol and cytoplasmic vacuoles rather than in the chloroplast [5]. These cytoplasmic LHCPs lacked the transit sequence, which indicated that they had visited the plastid stroma sufficiently for processing. Accumulation in vacuoles possibly occurred by transfer of chloroplast material in vesicles that budded from the plastid envelope, a process only observed in cryopreserved cells [6]. In the *y-1* mutant, LHCPs synthesized in excess of the ability of Chl to enable retention of the proteins in the plastid were retracted into the cytosol [5]. In the absence of Chl *b*, the lack of import of LHCPs was overcome when the algal cells were grown in medium containing acetate as a carbon source, which supported the synthesis of Chl *a* at a higher rate than in autotrophically grown cells [5,7]. Results of import assays with chloroplasts isolated from Chl *b*-less mutants of Arabidopsis differed depending on conditions, from no impairment [8] to complete lack of import of precursors of LHC apoproteins [9]. However, in the more stringent in vivo environment, chloroplasts from Chl *b*-less mutant plants were nearly devoid of the major LHCPs [8,9]. These results underline the critical role of Chl *b* in the import of LHCPs but also raise the question of whether another compound could replace Chl *b.* As noted below, protochlorophyllide (Pchlide) *a*, as the immediate precursor of Chlide *a*, has Lewis acid properties similar to those of Chlide *b*. Nevertheless, the contributions of Chl *b* to the assembly of LHCs are fundamental to achieving maximal capacity and efficiency of photosynthesis, and thus Chl *b* is one of the most essential compounds in the biosphere.

We previously [10] provided an outline of a proposed pathway of conversion of Chlide *a* to Chlide *b* based on evidence of a tyrosyl radical in Chlide *a* oxygenase (CAO), the enzyme that is required for this activity. Because of the importance of CAO, we provide herein a more extensive synthesis of additional, incisive information that brings an understanding of this pathway into sharper focus. We hope that the presentation of these data will provide a platform upon which detailed future experimental analyses can be performed.

### 1.2. Unanticipated Additional Functions of Chl b

Chl *b* exerts roles in addition to LHC assembly. In antisense lines of tobacco containing lower CAO expression than in the wild-type, reduced Chl *b* contents and electron transport rates through PSII and PSI were observed, which correlated with decreased carbon assimilation in plants grown under low or high light intensities [11]. Moreover, diminished flows of Chl precursors were noted, which could be due to a feedback loop by which Chl *b* controls expression of tetrapyrrole biosynthesis enzymes. Additional physiological effects can be inferred from studies on the *chlorina* mutants that contain defective CAO genes [12]. By contrast, up-regulation of Chl *b* biosynthesis by CAO over-expression enhanced the rates of electron transport and carbon assimilation in transgenic tobacco [13,14]. Chl *b* thus appears to have functions beyond those known in photosynthetic light-harvesting that are not yet fully characterized and deserve further work. Understanding CAO’s activity and role as the enzyme responsible for the synthesis of Chl *b* therefore has far-reaching implications for understanding plant physiology and chloroplast development.

## 2. Synthesis of Chl *b*

### 2.1. The Chlide a Oxygenase (CAO)-Catalyzed Reaction

The first committed precursor of Chl is glutamate 1-semialdehyde, the product of reduction in glutamyl-tRNA, which is then converted to 5-aminolevulinic acid by glutamate 1-semialdehyde 2,1-aminotransferase [15]. The pathway proceeds to Chlide *a* and then, in plants and green algae, extends further to Chlide *b*. These oxygenic photosynthetic organisms contain a gene that encodes a protein designated Chlide *a* oxygenase (CAO), which is required to convert the 7-methyl group of Chlide *a* to the 7-formyl group of Chl *b* [16,17]. The CAO protein includes sequences that generate a Rieske (2Fe-2S) cluster, a non-heme mononuclear iron center, and an unusual, conserved RYRxWR motif near the COOH-terminus. (The ‘x’ is ‘R’ in the protein in Chlamydomonas and ‘L’ in Arabidopsis.) Most studies of the reaction catalyzed by CAO have focused on the Rieske oxygenase character of the enzyme and largely ignored a possible role of the arginine-rich motif. Among the mechanisms for the synthesis of Chlide *b* from Chlide *a* that were proposed by Porra et al. [18], one included 7-methyl hydroperoxide as an intermediate, while another had 7-hydroxymethyl and -dihydroxymethyl derivatives as intermediates. Oster et al. [19] were the first to describe expression of the Arabidopsis CAO gene in *Escherichia coli* and presented data that were interpreted as evidence for two sequential monooxygenation reactions that produced 7-dihydroxymethyl Chl *a*, which by loss of a water molecule became Chl *b*.

Ito et al. [20] studied the metabolism of 7-hydroxymethyl Chlide *a* with isolated barley etioplasts but could not detect any conversion of the added substrate into the expected CAO product, that is, Chlide *b*. Instead, formation of Chlide *a* from 7-hydroxymethyl Chlide *a* was observed. This result was taken as evidence that barley etioplasts contain a 7-hydroxymethyl Chl *a* reductase normally involved in the breakdown of Chl(ide) *b* to Chl(ide) *a,* a known step in the Chl cycle [21]. Expression studies have shown that the CAO and 7-hydroxymethyl Chl *a* reductase genes in Arabidopsis are likewise expressed in etiolated plants such that at least some of the added 7-hydroxymethyl Chlide *a* should have been converted to Chlide *b,* which was not the case [20]. The recent cloning and X-ray structural analysis of 7-hydroxymethyl Chl *a* reductase from Arabidopsis [22] confirmed that 7-hydroxymethyl Chlide *a* is an intermediate in Chlide *b* breakdown, while leaving open its role in Chl *b* biosynthesis.

The sequential monooxygenation/hydroxylation pathway [18,19] was proposed prior to the detection of a unique, stable radical in CAO. The concept of a radical-based dioxygenation mechanism for the synthesis of Chlide *b* evolved from the discovery that the tyrosine (Y) residue in the RYRxWR motif exists as a free radical [23]. The tyrosyl radical is quenched by Chl *a* (possibly also by Chlide *a*), which indicates that a hydrogen atom (H•) was likely abstracted from Chl *a* to generate a 7-methyl radical (-CH_2_•). Molecular oxygen, a diradical, does not react with tyrosyl radicals—a spin-forbidden and thermodynamically unfavorable process [24]—but would react rapidly, as a barrierless process [25], with the 7-methyl radical to produce a peroxyl radical (CH_2_-O-O•). The tyrosine phenolic group would be re-oxidized by the peroxyl radical to restore the tyrosyl radical and produce the 7-methyl hydroperoxide (CH_2_-O-O-H) intermediate. Cleavage of the O-O bond would occur by attack of the Fe^II^ in the non-heme iron center. Elimination of Fe^II^ from the resulting ferryl group would produce Chlide *b*. The reaction requires transfer of an electron from NADPH, through the Rieske cluster, to reduce the non-heme iron to Fe^II^.

**Figure 2:**
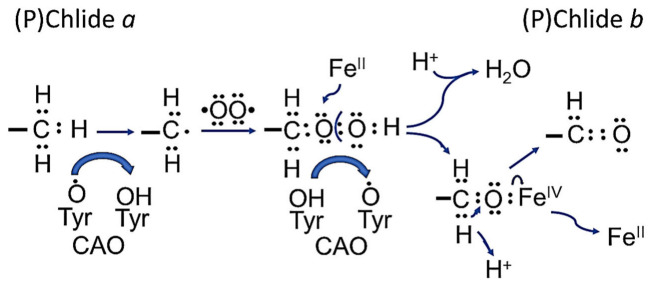
Diagrammatic presentation of the proposed mechanism of (Proto) Chlide *b* synthesis as described in the text. The first half of the pathway involves the interaction of the tyrosyl radical with the 7-methyl group of Chlide *a*. The second half involves the action of Fe^II^ in the non-heme iron center on oxygen-containing intermediates.

The radical species was identified as Tyr-422 in Chlamydomonas and Tyr-518 in Arabidopsis by the reaction of CAO with Na^125^I [23]. Evidence for the involvement of this tyrosine in the synthesis of Chlide *b* was obtained by replacement of Tyr-518 in CAO from Arabidopsis with alanine by site-directed mutagenesis, which abolished activity in assays performed in vitro [9,10]. The mechanism of the synthesis of Chlide *b* that we propose follows logically and stepwise from the abstraction of a hydrogen atom from Chlide *a* by the tyrosyl radical to the release of the final product, as shown in Figure 2.

### 2.2. Effect of Site-Directed Mutagenesis in the Rieske Cluster and Non-Heme Iron Center

CAO from different sources (plants and green algae) feature the presence of a Rieske (2Fe-2S) cluster and a non-heme iron (Fe) center. Transfer of electrons needed to oxygenate the methyl group on the chlorin (or porphyrin) ring is thought to occur from ferredoxin or thioredoxin to the Rieske (2Fe-2S) cluster and then to the mononuclear non-heme iron binding site in the C-domain of the protein [26,27]. Dey et al. [27] proposed a model of CAO structure in which four conserved amino acids—C262, H264, C281, and H284 (for the Arabidopsis CAO)—interacted with the Rieske (2Fe-2S) cluster. Two histidine residues bind one iron, whereas the other iron ion is bound via the cysteine residues. Docking studies of the Fe^II^/Fe^III^ in the non-heme iron center suggested that four other conserved residues—N361, H367, H372, and D487—are responsible for interaction with the non-heme iron near the Ch(ide) *a* binding site.

Because direct experimental evidence for the involvement of these different residues in CAO’s catalytic mechanism did not yet exist, in vitro mutagenesis studies were carried out on the CAO enzyme of Arabidopsis. Table 1 summarizes the results of replacing C262, H264, C281, and H284 with alanine individually or in combination with the other residues. Double, triple, or quadruple mutations increasingly abolished CAO activity. Similarly, single, double, triple, or quadruple replacements of N361, H367, H372, and D487 in the non-heme iron center with alanine residues caused progressively decreasing CAO activity to zero (Table 1). These results are consistent with studies on *chlorina* mutants of barley containing alterations in the CAO gene. Mueller et al. [12] identified a series of missense mutations in the *CAO* gene of which *clo-f2.102* (leading to the amino acid exchange D495N in barley/D487N in Arabidopsis), *clo-f2.103* (G280D) and *clo-f2.133* (A376V/A368V) are most relevant because they are conserved residues, such as those of the Rieske (2Fe-2S) cluster (*clo-f2.103*) or the non-hme iron binding center (*clo-f2.102* and *clo-f2.133*). These mutants were either devoid of Chl *b* (*clo-f2.102* and *clo-f2.103*) or expressed lowered levels of this pigment (*clo-f2.133*). Our in vitro mutagenesis studies confirm and extend these findings and thus support the overall structural model proposed by Dey et al. [27]. Collectively, the results shown in Table 1 demonstrate the essential role of the Rieske cluster and non-heme iron center in catalysis, but they do not necessarily identify the catalytic site.

### 2.3. Predicting a Putative Catalytic Site of CAO

**Figure 3:**
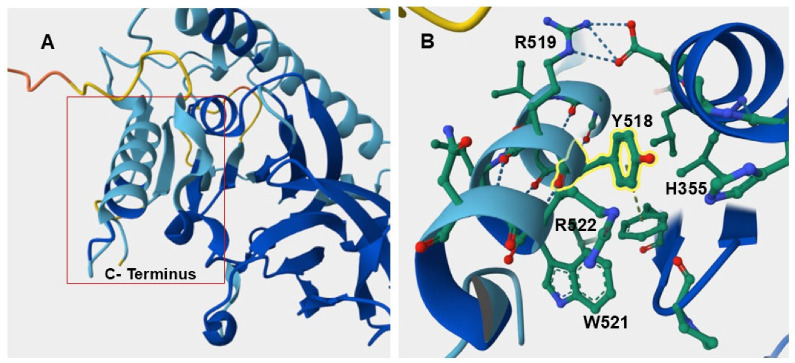
AlphaFold prediction of the structure of CAO from Arabidopsis. (**A**) A portion of the C-domain of the enzyme is shown with the predicted catalytic center boxed. (**B**) The portion of the protein boxed in (**A**) is expanded in (**B**) to show the tyrosine (Y518) (highlighted) that bears the free radical and other amino acids within the catalytic center. The per-residue confidence score for the protein was 76.81, with scores for the dark blue segments greater than 90. Adapted from UniProt, entry Q9MBA1.

Because substitution of a single residue—Tyr-518 (Y518)—with alanine abolished activity of CAO from Arabidopsis [10], this position in the enzyme was assumed to be the core of the catalytic site. The tyrosyl radical seems most likely to engage the substrate, as described in Figure 2, with electron transfer into the catalytic site occurring through the Rieske cluster and the non-heme iron center. In the absence of a crystal structure for CAO, we searched the structure predicted by AlphaFold for a model of the putative catalytic site (Figure 3). The predicted structure of the Arabidopsis CAO projects the tyrosyl radical (Y518) sidechain into a cleft between two α-helices. The base of the Y518-containing helix is a flexible, proline-containing strand that would allow the cleft to expand to accommodate the substrate, whether presented as a monomer or a dimer (see below). In this model, a conserved histidine (H355) is located in a smaller helix that forms the opposite wall of the presumed catalytic site. The imidazole sidechain of H355 possibly forms a coordination bond with the Mg^2+^ of Chlide *a* to position the substrate. We hypothesize that binding could be enhanced further by interaction of the 17^3^-carboxyl group of the sidechain in Chlide *a* with the guanidinium group of arginine (R522) and by nonpolar van der Waals forces with the sidechain of tryptophan (W521). These residues would be expected to contribute significantly to the specificity of binding of the substrate. For the reasons outlined below, the product of the reaction, Chlide *b*, would no longer bind H355 and thus leave the binding site. Conformational changes during catalysis and formation of the electronegative formyl group increase exposure of the Mg^2+^ and would trigger the release of the product from the enzyme (see below under Section 3.3).

## 3. Characteristics of the CAO-Catalyzed Reaction

### 3.1. Dissociation Energy for Oxygen Bond Cleavage

A feature of both proposed reaction mechanisms—monooxygenation vs. dioxygenation—of CAO is cleavage of the bond between oxygen atoms by reduced iron (Fe^II^) in the non-heme iron center. In the hydroxylation pathway proposed by Oster et al. [19] and studied in detail by Liu et al. [26], bond cleavage is thought to occur at the molecular oxygen stage to generate reactive monooxygen-iron complexes. The dissociation energy of the O-O bond in molecular oxygen is 497 kJ/mol [28], and because a molecule of oxygen is used for each hydroxylation reaction, the minimal energy required to achieve the 7-dihydroxymethyl intermediate would be nearly 1000 kJ/mol. In the radical-based pathway, bond cleavage occurs in the hydroperoxide group, with a dissociation energy of the O-O bond of about 190 kJ/mol [29]. Thus, considering only cleavage of this bond, the energy barrier for the dihydroxylation mechanism would be much greater than for the radical-mediated pathway.

### 3.2. Consideration of Redox Potentials

Evidence from in vitro studies suggests that CAO can catalyze at a low rate the conversion of monomeric Chlide *a* to Chlide *b* [19,26] but not Pchlide *a* to Pchlide *b* [9]. Data reviewed by Fuhrhop [30] indicated that the oxidation potential of Mg octaethylporphyrin is approximately 4-fold higher than that of Mg octaethylchlorin. By analogy, the redox potential of Pchlide *a,* a porphyrin, is considerably higher than that of Chlide *a*, a chlorin. It is attractive to hypothesize that the rate of the reaction of Chlide *a* to Chlide *b* with recombinant CAO was possibly low because of the relatively small difference in redox potentials between Chlide *a* (E^1^ = +0.84 V) and the tyrosyl radical, which in proteins is +0.94 V at pH 7 [31,32]. However, adjacent arginine residues lower the redox potential of the tyrosyl radical to about +0.86 V [32]. Thus, whereas the arginine residues (R) in the RYRxWR motif of CAO may stabilize the tyrosyl radical, they possibly also lower the rate of the reaction.

Dimerization of Chl *a* causes a negative shift in its redox potential from +0.84 V to +0.71 V in an ionic organic solvent or to as low as +0.5 V in an aqueous environment [33,34]. We propose that dimerization of Chlide *a* compensates for a lower redox potential of the tyrosyl radical in the arginine-rich motif. The ensuing greater difference between the potential of a Chlide *a* dimer and that of the tyrosyl radical would significantly increase the rate of the reaction catalyzed by CAO. Chlide *a* dimerization generally occurs at concentrations in the micro- to millimolar range, depending on the environment. Interestingly, formation of Chl *a* dimers is facilitated by the lipid matrix of thylakoid membranes, in which the antiparallel, cofacial configuration of the dimeric chlorin macrocycle is more compact and thermodynamically favored [35,36].

### 3.3. Lewis Acid Properties

**Figure 4:**
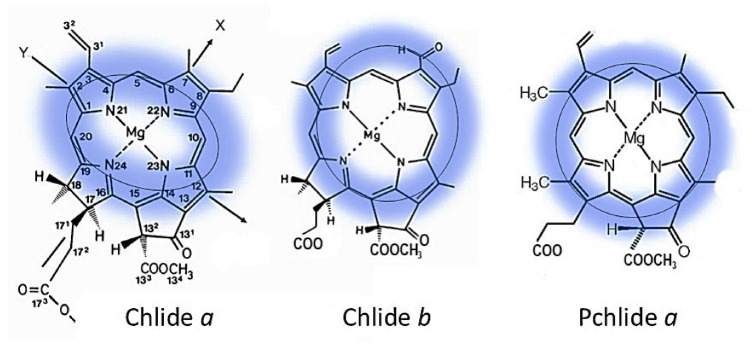
Representation of the π-electron cloud in blue shade. The numbering system and molecular axes are shown in the structure of Chl *a*. Chl *b* differs from Chl *a* by containing a formyl group rather than a methyl group at position 7. Pchlide *a* contains a double bond between carbons 17 and 18. Chls lacking the phytol sidechain are designated by the ‘-ide’ ending.

The introduction of the electronegative oxygen by the formation of the 7-formyl group alters the type of coordination bond formed by the central Mg^2+^ atom. As illustrated in Figure 4, the Mg^2+^ of Chlide *a* is partially shielded by the macrocycle’s elliptical π-electron cloud along the molecular *Y* axis. The partial transfer of electrons from the imidazole group of histidine to Mg^2+^ during formation of an axial coordination bond by orbital interactions results in a partial positive charge on the imidazole group that is attracted by the π-electron cloud [3]. In Chlide *b*, the formyl oxygen pulls the π-electron density ‘northward’ along the *X* axis and increases exposure of the Mg^2+^ atom. In a similar fashion, the π-electron density of Pchlide *a* is expanded ‘southward’ from N24 and N23 along the *X* axis toward the periphery of the molecule to include the C17-C18 double bond. Exposure of the positive charge on Mg^2+^ repels a partial positive charge on the imidazole group but favors formation of a stable, electrostatic coordination bond with the negative end of an oxygen dipole [3]. A histidine residue is expected to reside in the substrate binding pocket of CAO, whose imidazole group is a soft Lewis base and readily forms a coordination bond with the Mg^2+^ of Chlide *a*, as demonstrated previously [2,37]. In contrast, the Mg^2+^ in Chlide *b* is a stronger Lewis acid and forms bonds with a carbonyl oxygen, a strong Lewis base. Thus, whereas Chlide *a* can form a coordination bond with the imidazole group, neither Chlide *b* nor Pchlide *a* would form a bond with histidine in the substrate binding site; the axial coordination position would likely then be filled by a water molecule. Along with lowering the redox potential of Pchlide *a*, *m*-phenanthroline possibly softens the Lewis acid strength of the central Mg^2+^, which allows Pchlide *a* to form a coordination bond with the active site histidine and thus serve as a substrate.

### 3.4. In Vitro Studies with Native CAO

A membrane fraction, purified from cells of the *y-1* mutant of *Chlamydomonas reinhardtii,* was unable to convert Pchlide *a* that was bound in the membrane to Pchlide *b*. However, CAO in the membrane fraction rapidly converted the Pchlide *a* to Pchlide *b* in the dark when the polyaromatic *m*-phenanthroline was added to the in vitro assay [10,38]. The requirement of phenanthroline for the conversion of Pchlide *a* to Pchlide *b* may indicate that a direct interaction of this ‘cofactor’ with the substrate was required to lower its redox potential sufficiently for the reaction to occur [38,39]. Pchlide *b* was further converted to Chlide *b* by reduction of the C17-C18 double bond, a reaction facilitated by withdrawal of electron density from the double bond across the *X* axis of the molecule by the electronegative 7-formyl oxygen. In assays exposed to the light, Pchlide *a* was rapidly reduced to Chlide *a* by Pchlide *a* oxidoreductase in the membrane fraction, but Chlide *b* was not detected. It is possible that the high rate of synthesis of Chlide *a* in vivo is sufficient, in contrast to in vitro conditions, to provide a functional, dimeric substrate for CAO. Chlide *a* then possibly becomes both substrate and ‘cofactor’ for CAO during chloroplast development. These data suggest a mechanism for the regulation of the synthesis of Chlide *b* by the availability of Chlide *a*.

## 4. Conclusions

The evidence summarized in this article sheds new light on the structure and function of the Rieske-type enzyme, CAO. Its unusual substrate binding and reaction mechanism provide fresh fuel to the discussion on the role of this enzyme in plants and green algae. The effect of *m*-phenanthroline on the reaction catalyzed by CAO in Chlamydomonas is particularly instructive, because it suggests that dimerization is required to decrease the redox potential of the native substrate. Pchlide *a* is not converted to Pchlide *b* in the absence of *m*-phenanthroline. In the light, the activity of the light-dependent Pchlide *a* oxidoreductase may outpace that of CAO, which would then determine that Chlide *b* is formed from Chlide *a*. During normal chloroplast development, the rate of Chlide *b* synthesis is possibly increased by dimerization of Chlide *a* as the substrate. Studies of cofacial dimers of methyl Chlide *a,* in which the macrocycles are oriented 180° from each other, revealed that the major stabilizing factors are π-π stacking and formation of a coordination bond between the Mg^2+^ of one and a carbonyl oxygen of the other [35,36]. In this configuration, the dimer is more compact than in the parallel orientation [36], which would allow it to slip into the predicted CAO catalytic site more readily, with the 7-methyl group of only one of the Chlide *a* molecules near the tyrosyl radical.

AlphaFold-based structural modeling predicts the presence of a conserved, unique substrate binding cleft in which a tyrosine (Y518) residue is near arginine (R522 and R519), tryptophan (W521), and histidine (H355) residues. We propose that the tyrosyl radical initiates a pathway that includes 7-methyl hydroperoxide as an intermediate in the transformation of the C7-methyl group of Chlide *a* into the formyl group of Chlide *b*. During normal chloroplast development, the high rate of the CAO-catalyzed reaction suggests that the substrate is a homodimer, in which one of the Chlide *a* molecules is converted to Chlide *b* while the other acts as a cofactor. Interestingly, this interaction may be reflected in the kinetics of esterification of Chlide *a* and Chlide *b* with geranylgeranyl pyrophosphate and sequential reduction to the phytyl sidechain, which are virtually the same during chloroplast development for both Chls, Chl *a* and Chl *b* [7].

In this article, we have synthesized relevant known information that bears on the mechanism of Chl *b* synthesis in plants and green algae. Our conclusions confirm and clarify early observations on the synthesis of Chl *b* [10]. A full understanding of the pathway catalyzed by the important enzyme CAO, including our proposal of a radical-mediated reaction, requires more detailed experimental analyses. Further studies are needed to resolve the 3D-structure of CAO and to test the proposed reaction mechanism. The information in this article should provide a platform on which to build.

## Figures and Tables

**Table 1:** Activity of purified recombinant Arabidopsis Chlide *a* oxygenase mutant proteins.

Conserved Residues in the Rieske [2Fe-2S] Cluster	Chlide a Oxygenase Activity (nkat/mg CAO Protein)	
Single substitutions		
C262A	1.40 ± 0.13	(55.5%)
H264A	1.37 ± 0.12	(54.4%)
C281A	1.38 ± 0.15	(54.8%)
H284A	1.42 ± 0.18	(56.3%)
Double substitutions		
C262A + H264A	0.22 ± 0.02	(8.7%)
C262A + C281A	0.24 ± 0.02	(9.5%)
C281A + H284A	0.18 ± 0.02	(7.1%)
Triple substitutions		
C262A + H264A + C281A	n.d.	(0%)
C262A + H264A + H284A	n.d.	(0%)
Quadruple substitution		
C262A + H264A + C281A + H284A	n.d.	(0%)
Conserved residues in the non-heme iron center		
Single substitutions		
N361A	1.20 ± 0.14	(47.6%)
H367A	1.28 ± 0.20	(50.8%)
H372A	1.16 ± 0.12	(46.0%)
D487A	1.02 ± 0.10	(40.5%)
Double substitutions		
N361A + H367A	0.40 ± 0.03	(15.9%)
N361A + H372A	0.20 ± 0.02	(7.9%)
N361A + D487A	0.18 ± 0.01	(7.1%)
Triple substitutions		
N361A + H367A + H372A	n.d.	(0%)
N361A + H367A + D487A	n.d.	(0%)
Quadruple substitution		
N361A + H367A + H372A + D487A	n.d.	(0%)

The indicated amino acid substitutions were introduced into the Arabidopsis CAO gene by site-directed mutagenesis using published commercial protocols. The respective plasmid clones were expressed in *E. coli* as CAO-(His)_6_ mutant proteins containing the amino acid replacements and purified from bacterial extracts by affinity chromatography on Ni-NTA agarose. Activity assays in turn were carried out as described previously [9], using Chlide *a* as substrate and glucose-6-phosphate, NADPH, glucose-6-phosphate dehydrogenase, ferredoxin, and ferredoxin:NADPH oxidoreductase (FNR) as supplements. CAO activities were determined in three replicate samples. For the wild-type enzyme, a CAO activity of 2.52 ± 0.18 nkat/mg protein was determined. Numbers in parentheses define the percentage decrease in CAO activity relative to the wild-type activity. n.d. indicates no detectable CAO activity.

## Data Availability

The original contributions presented in this study are included in the article. Further inquiries can be directed to the corresponding authors.
